# IgA dominates the early neutralizing antibody response to SARS-CoV-2

**DOI:** 10.1126/scitranslmed.abd2223

**Published:** 2020-12-07

**Authors:** Delphine Sterlin, Alexis Mathian, Makoto Miyara, Audrey Mohr, François Anna, Laetitia Claër, Paul Quentric, Jehane Fadlallah, Hervé Devilliers, Pascale Ghillani, Cary Gunn, Rick Hockett, Sasi Mudumba, Amélie Guihot, Charles-Edouard Luyt, Julien Mayaux, Alexandra Beurton, Salma Fourati, Timothée Bruel, Olivier Schwartz, Jean-Marc Lacorte, Hans Yssel, Christophe Parizot, Karim Dorgham, Pierre Charneau, Zahir Amoura, Guy Gorochov

**Affiliations:** 1 Sorbonne Université, Inserm, Centre d’Immunologie et des Maladies Infectieuses (CIMI-Paris), 91 boulevard de l’Hôpital, 75013 Paris, France.; 2 Département d’Immunologie, Assistance Publique-Hôpitaux de Paris (AP-HP), Hôpital Pitié-Salpêtrière, 83 boulevard de l’Hôpital, 75013 Paris, France.; 3 Unit of Antibodies in Therapy and Pathology, Institut Pasteur, UMR1222, Inserm, 25-28 Rue du Dr Roux, 75015 Paris, France.; 4 Service de Médecine Interne 2, Institut E3M, AP-HP, Hôpital Pitié-Salpêtrière, 83 boulevard de l’Hôpital, 75013 Paris, France.; 5 Unité de Virologie Moléculaire et Vaccinologie, Institut Pasteur, 25-28 Rue du Dr Roux, 75015 Paris, France.; 6 Theravectys, Institut Pasteur, 25-28 Rue du Dr Roux, 75015 Paris, France.; 7 Centre Hospitalier Universitaire de Dijon, Hôpital François Mitterrand, service de médecine interne et maladies systémiques (médecine interne 2) et Centre d’Investigation Clinique, Inserm CIC-EC 1432, 3 rue du FBG Raines, 21000 Dijon, France.; 8 Genalyte Inc., 10520 Wateridge Circle, San Diego, CA 92121, USA.; 9 Service de Médecine Intensive Réanimation, Institut de Cardiologie, APHP, Sorbonne-Université, Hôpital Pitié-Salpêtrière, 83 boulevard de l’Hôpital, 75013 Paris, France.; 10 Sorbonne Université, INSERM, UMRS 1166-ICAN Institute of Cardiometabolism and Nutrition, 91 boulevard de l’Hôpital, 75013 Paris, France.; 11 Service de Médecine Intensive–Réanimation et Pneumologie, APHP, Hôpital Pitié-Salpêtrière, 83 boulevard de l’Hôpital, 75013 Paris, France.; 12 Sorbonne Université, Inserm UMRS Neurophysiologie respiratoire expérimentale et clinique, AP-HP, 91 boulevard de l’Hôpital, 75013 Paris, France.; 13 Service de Biochimie Endocrinienne et Oncologique, AP-HP, Hôpital Pitié-Salpêtrière, 83 boulevard de l’Hôpital, 75013 Paris, France.; 14 Inserm UMR1149, Centre de Recherche sur l’Inflammation Paris Montmartre (CRI), 16 rue Henri Huchard, 75890 Paris, France.; 15 Virus and Immunity Unit, Department of Virology, Institut Pasteur, 25-28 Rue du Dr Roux, 75015 Paris, France.; 16 CNRS-UMR3569, Institut Pasteur, 25-28 Rue du Dr Roux, 75015 Paris, France.; 17 Vaccine Research Institute, 51 avenue du Maréchal de Lattre de Tassigny, 94000 Créteil, France.

## Abstract

Humoral immune responses are typically characterized by primary IgM antibody responses followed by secondary antibody responses associated with immune memory and composed of IgG, IgA, and IgE. Here, we measured acute humoral responses to SARS-CoV-2, including the frequency of antibody-secreting cells and the presence of SARS-CoV-2–specific neutralizing antibodies in the serum, saliva, and bronchoalveolar fluid of 159 patients with COVID-19. Early SARS-CoV-2–specific humoral responses were dominated by IgA antibodies. Peripheral expansion of IgA plasmablasts with mucosal homing potential was detected shortly after the onset of symptoms and peaked during the third week of the disease. The virus-specific antibody responses included IgG, IgM, and IgA, but IgA contributed to virus neutralization to a greater extent compared with IgG. Specific IgA serum concentrations decreased notably 1 month after the onset of symptoms, but neutralizing IgA remained detectable in saliva for a longer time (days 49 to 73 post-symptoms). These results represent a critical observation given the emerging information as to the types of antibodies associated with optimal protection against reinfection and whether vaccine regimens should consider targeting a potent but potentially short-lived IgA response.

## Early action of IgA

Humoral immune responses play a critical role in protecting individuals against SARS-CoV-2 infection, particularly through the activity of neutralizing antibodies. Sterlin *et al*. measured humoral immune responses in the serum, saliva, and bronchoalveolar lavage fluid of SARS-CoV-2–infected patients who experienced a range of COVID-19 disease severity. IgA antibodies dominated the early SARS-CoV-2–specific antibody response compared with IgG and IgM concentrations in these fluids and was associated with expansion of IgA plasmablasts with mucosal homing characteristics. IgA serum concentrations peaked 3 weeks after symptom onset but persisted for several more weeks in saliva, and serum IgA was more potent than IgG in neutralizing SARS-CoV-2. These findings highlight the potential role of IgA during early SARS-CoV-2 infection.

## Teaser

Early specific antibody responses against SARS-CoV-2 include IgG, IgM, and IgA, but IgA may neutralize virus and control infection to a larger extent.

## INTRODUCTION

In December 2019, a novel coronavirus named SARS-CoV-2 (severe acute respiratory syndrome coronavirus 2) was identified as the cause of an acute respiratory disease known as coronavirus disease 2019 (COVID-19). This enveloped positive-sense RNA virus is a member of the betacoronavirus and spread worldwide with an unprecedented speed compared with the dissemination of SARS-CoV in 2003 and Middle East respiratory syndrome–related coronavirus virus (MERS-CoV) in 2012 (*1*). Recent reports indicate that SARS-CoV-2 elicits robust humoral immune responses, including production of virus-specific antibodies of the immunoglobulin M (IgM), IgG, and IgA isotypes. Patients have been shown to achieve seroconversion and produce detectable antibodies within 20 days of symptom onset, although the kinetics of IgM and IgG production are variable (*2*–*4*).

Secretory IgA plays a crucial role in protecting mucosal surfaces against pathogens by neutralizing respiratory viruses or impeding their attachment to epithelial cells (*5*–*8*). Influenza-specific IgA has been shown to be more effective in preventing infections in mice and humans compared with influenza-specific IgG, and elevated IgA serum levels have been correlated with influenza vaccine efficacy (*9*–*11*). IgA may also play an important role in SARS-CoV infection. In mice, intranasal vaccination with SARS-CoV proteins induces localized and systemic virus-specific IgA responses and provides better protection against SARS-CoV challenge compared with intramuscular delivery, suggesting that mucosal-induced IgA is protective (*12*). A recently reported intervention based on an intranasal immunization with a MERS-derived vaccine confirmed a beneficial role of IgA (*13*). However, the nature of the virus-specific IgA response against SARS-CoV-2 infection in humans remains poorly understood.

We tracked antibody-secreting cells, characterized here as plasmablasts, in the blood of SARS-CoV-2–infected patients. We measured specific antibody titers longitudinally in serum and compared the neutralizing capacities of purified serum monomeric IgA and IgG. Lastly, we studied the neutralization potential of mucosal antibodies present in lower respiratory tract pulmonary secretions and saliva. Our results show that human IgA antibodies are often detectable before the appearance of SARS-CoV-2–specific IgG and suggest a role for IgA antibodies in early virus neutralization.

## RESULTS

### Circulating plasmablasts preferentially express IgA1

**Fig. 1:**
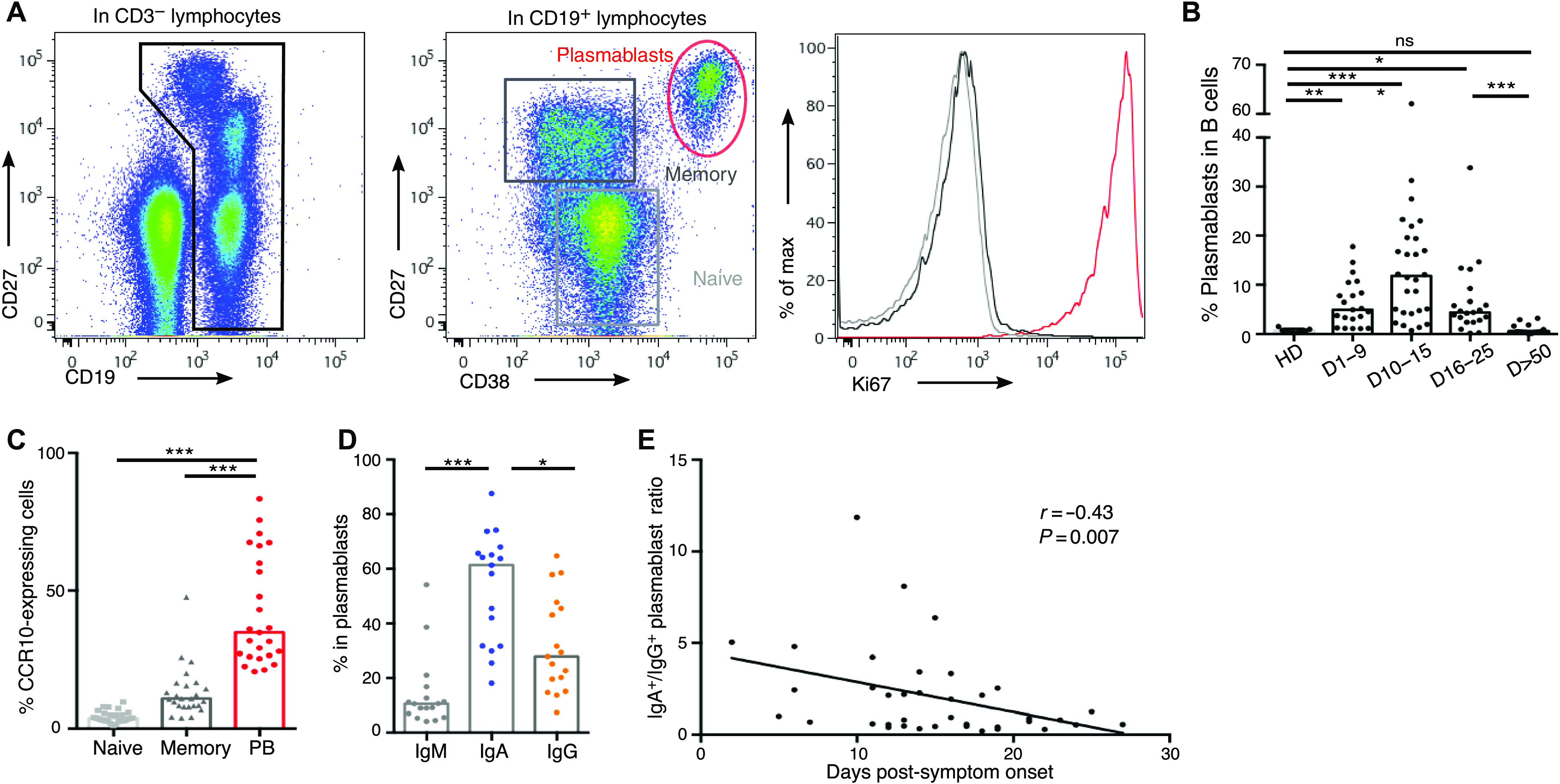
Plasmablast dynamics after SARS-CoV-2 infection. (**A**) Representative flow cytometry analysis of B cell subpopulations in the blood of SARS-CoV-2–infected patients. Doublets and dead cells were excluded before CD3^−^CD19^+^ gating. Plasmablasts are defined as Ki67^+^CD19^low^CD27^high^CD38^high^ cells, memory B cells as Ki67^−^CD19^+^CD27^+^IgD^−^, and naive B cells as Ki67^−^CD19^+^CD27^−^IgD^+^ cells. (**B**) Plasmablast frequency in B cell compartment in blood of SARS-CoV-2–infected patients (*n* = 38, clinical characteristics in table S1) compared with healthy donors (HD; *n* = 9). Histograms represent medians. *P* values were calculated using Dunn’s multiple comparison test (*P < 0.05, **P < 0.01, and ***P < 0.001). ns, not significant. (**C**) Flow cytometry analysis of CCR10 expression in B cell subpopulations in blood of SARS-CoV-2–infected patients (*n* = 25). Samples used in this analysis were collected from day 3 to day 27 after symptom onset. Histograms represent medians. *P* values were calculated using Wilcoxon test (***P < 0.001). (**D**) Intracellular antibody expression in circulating plasmablasts in blood of SARS-CoV-2–infected patients (*n* = 17) using flow cytometry. Samples used in this analysis were collected from days 2 to 23 after symptom onset. Histograms represent medians. *P* values were calculated using Dunn’s multiple comparison test (*P < 0.05 and ***P < 0.001). (**E**) Intracellular IgA versus IgG expression in plasmablasts according to disease duration. Each dot represents one patient. Nonparametric Spearman correlation was calculated.

The rapid and transient appearance of plasmablasts in peripheral blood is a common feature of the acute phase of viral infections (*14*). We monitored phenotypic changes of B cells longitudinally in the blood of 38 SARS-CoV-2–infected patients (table S1) using flow cytometry. Plasmablasts are immature antibody-secreting cells, defined here as proliferating cell-cycling Ki67^+^CD19^low^CD27^high^CD38^high^ cells (Fig. 1A and fig. S1A). The proportion of plasmablasts in the B cell compartment increased significantly at days 1 to 9 after the onset of symptoms (median[minimum to maximum]%; 4.9[1.1 to 17.8]% *n* = 21 versus 0.5[0.1 to 1.5]% in healthy donors *n* = 9; *P* = 0.0068; Fig. 1B) peaked between days 10 and 15 (11.8[0.7 to 62.1]%, *n* = 28; Fig. 1B), and then decreased (4.4[0.2 to 33.8]%, between days 16 and 25, *n* = 21; 0.5[0.1 to 3.2]%, after day 50, *n* = 14; Fig. 1B). Longitudinal follow-up in seven patients also confirmed the transient nature of plasmablast expansion during acute viral infection (fig. S1B).

We analyzed circulating plasmablasts for surface expression of CCR10, a chemokine receptor involved in the migration of immune cells to mucosal sites, especially the lung (*15*, *16*). Less than 10% of memory and naive B cells, but about 40% of detected plasmablasts, were CCR10^+^ (3.8[1.2 to 9.6]% in naive B cells versus 10.9[4.1 to 47.7]% in memory B cells versus 34.9[20.7 to 83.4]% in plasmablasts; *n* = 25; Fig. 1C), suggesting a potential lung tissue tropism of the latter. Analysis of the early phase of the immune response revealed only a minor population of plasmablasts that produced IgM, as measured by intracellular staining (10.5[4.2 to 54.1]% IgM^+^ plasmablasts, *n* = 17; Fig. 1D). In contrast, most plasmablasts expressed IgA (61.4[18.1 to 87.6]% IgA^+^ plasmablasts versus 27.9[7.4 to 64.8]% IgG^+^ plasmablasts, *n* = 17; Fig. 1D), a feature consistent with plasmablasts that are found at mucosal sites. Intracellular IgA subclass identification showed higher frequencies of IgA1-expressing plasmablasts, as compared with IgA2 (66[26.8 to 88.5]% IgA1^+^ versus 31.6[3.7 to 70.8]% IgA2^+^ in IgA^+^ plasmablasts, *n* = 13; fig. S1, C and D). This first wave of circulating IgA-expressing plasmablasts, peaking between days 10 and 15, was followed by a second wave of IgG-expressing cells that were more dominant by day 22 after the onset of symptoms (Fig. 1E and fig. S1, E and F). The majority of IgA^+^ plasmablasts expressed CCR10, but this chemokine receptor was expressed by a minority of IgG^+^ plasmablasts (60.5[37.6 to 92.6] versus 23.3[3.2 to 78]% CCR10^+^, *n* = 15; fig. S1G), suggesting that the latter may occupy a different niche, such as the bone marrow. The frequency of peripheral IgM-expressing plasmablasts did not vary significantly at early time points (fig. S1H) and only marginally at later time points (fig. S1I).

In a recent study that characterized the immune response of a COVID-19 patient, the induction of T follicular helper (T_FH_) cells was reported to occur concomitantly with that of plasmablasts (*17*). To evaluate a potential germinal center origin of the plasmablast wave observed in our patients, we tracked CD4^+^CXCR5^+^PD1^+/−^ T_FH_ cells longitudinally in their blood. We found no significant increase in the frequency of T_FH_ subsets in COVID-19 patients, as compared to healthy donors, at any of the analyzed time points (fig. S2, A and B). The frequency of neither activated (CD4^+^CXCR5^+^PD1^+^) nor latent (CD4^+^CXCR5^+^PD1^−^) T_FH_ cells was found to correlate with that of plasmablasts (fig. S2C). Together, these results point toward an early humoral response to SARS-CoV-2 dominated by IgA-expressing plasmablasts that have a phenotype consistent with plasmablasts found at mucosal sites.

### Early SARS-CoV-2–specific IgA detection

We assessed the prevalence of IgG, IgA, and IgM antibodies recognizing the SARS-CoV-2 full-length nucleocapsid protein (NC) or spike receptor-binding domain (RBD) in serum samples from 132 infected patients (tables S2 and S3) using a photonic ring immunoassay, which can measure the level of antibodies to multiple antigens simultaneously (fig. S3) (*18*, *19*).

**Fig. 2:**
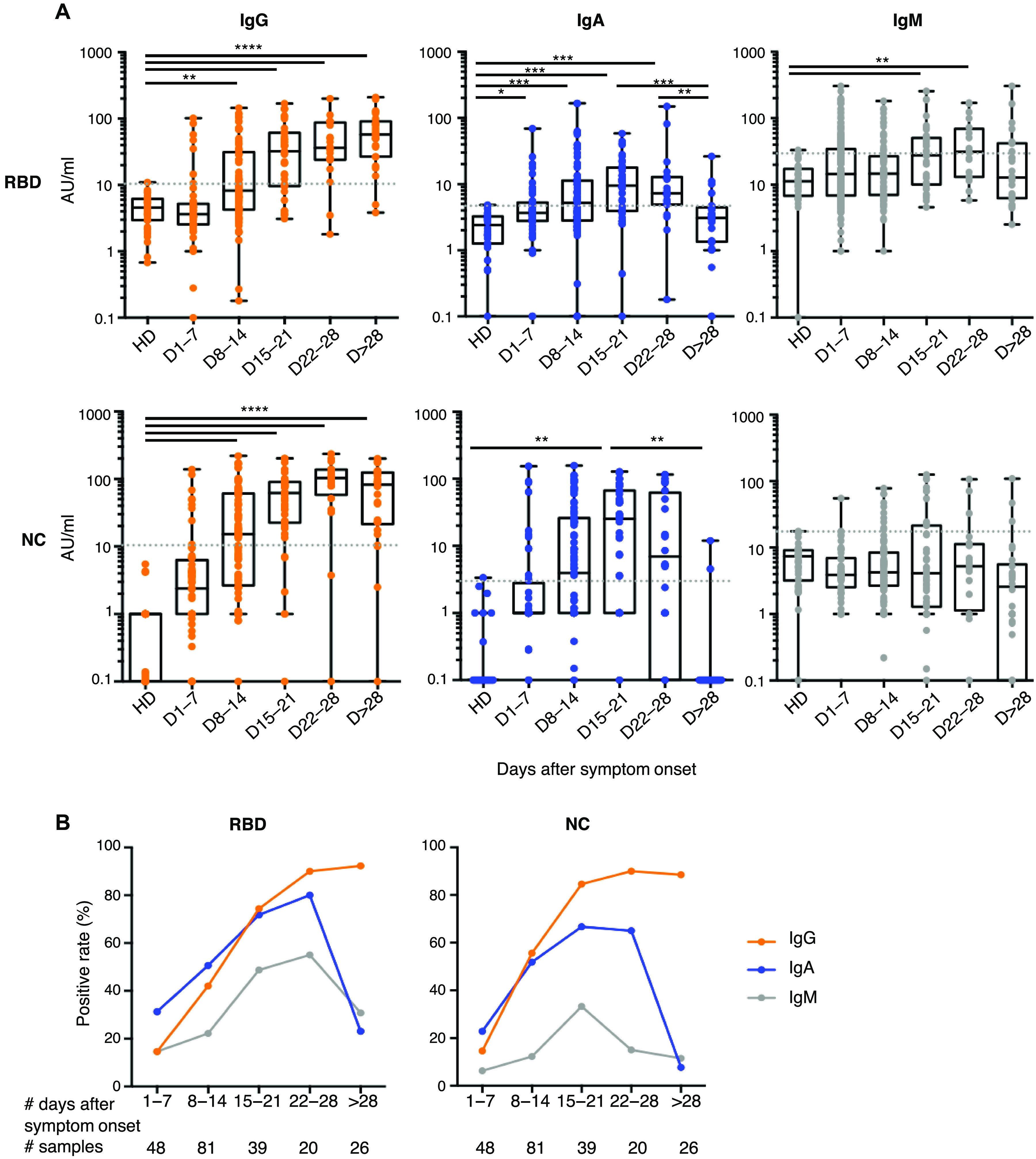
Antibody response kinetics to SARS-CoV-2 proteins. (**A**) Specific IgG, IgA, and IgM against spike-1 receptor-binding domain (RBD) and nucleocapsid protein (NC) were measured using photonic ring immunoassay in 132 patients (clinical characteristics detailed in tables S2 and S3). Antibody levels are expressed as arbitrary units/ml (AU/ml). Cutoff lines are represented as gray dotted lines. The boxplots show medians (middle line) and first and third quartiles, and the whiskers indicate minimal and maximal values. *P* value was calculated using Dunn’s multiple comparison test (*P < 0.05, **P < 0.01, ***P < 0.001, and ****P < 0.0001). (**B**) Positive rates of specific serum IgG, IgA, and IgM in 132 patients at different times after symptom onset, from days 1 to 78.

Data presented in Fig. 2B suggested that serum anti-RBD IgA might be detected earlier than anti-RBD IgG. In the subset of patients monitored at the very early time points after disease onset (first 7 days post-symptoms, *n* = 48), anti-RBD IgA and IgG were detected in 15 and 7 samples, respectively, at concentrations that did not reach statistical significance (positive samples: 31% IgA versus 15% IgG, *P* = 0.052; calculated from data presented in Fig. 2, A and B, and fig. S4A), whereas the positive rates of anti-NC were similar regardless of isotype (positive samples: 23% IgA versus 15% IgG, *P* = 0.43; Fig. 2B). However, in a subset of patients monitored at multiple time points (*n* = 38), time to positivity was significantly shorter for anti-RBD IgA than IgG (12[3 to 24] versus 15[8 to 24] days, *P* = 0.03; fig. S4B). IgM is typically considered a marker of acute infection, but anti-RBD IgM was detected only in 7 of these 48 early samples (Fig. 2A). Moreover, anti-NC IgM remained undetectable in all samples except one. These results suggest that serum anti-RBD IgA is likely to be detected earlier than anti-RBD IgG.

The proportion of patients with detectable anti-RBD IgG increased until hitting a plateau around the fourth week post-symptom onset (positive samples: 15%, days 1 to 7; 42%, days 8 to 14; 74%, days 15 to 21; 90%, days 22 to 28; and 92%, day >28; Fig. 2, A and B). In contrast, the frequency of patients with anti-RBD IgA peaked around day 22 (positive samples: 31%, days 1 to 7; 51%, days 8 to 14; 72%, days 15 to 21; and 80%, days 22 to 28; Fig. 2, A and B) and then decreased by day 28 (positive samples: 23%, day >28; Fig. 2, A and B). Following similar kinetics with respect to the appearance of anti-RBD antibodies, the proportion of patients with detectable anti-NC IgG remained stable around the fourth week post-symptom onset (positive samples: 15%, days 1 to 7; 56%, days 8 to 14; 85%, days 15 to 21; 90%, days 22 to 28; and 89%, day >28; Fig. 2, A and B), whereas anti-NC IgA quickly disappeared and were no longer detectable in most patients 1 month after disease onset (positive samples: 23%, days 1 to 7; 52%, days 8 to 14; 67%, days 15 to 21; 65%, days 22 to 28; and 8%, day >28; Fig. 2, A and B). In two patients who recovered from COVID-19, no specific antibodies were detected at days 32 and 47 (Fig. 2A).

Both anti-RBD and anti-NC IgG titers increased over time [anti-RBD arbitrary units (AU)/ml: 3.6[0.1 to 102.1], days 1 to 7; 8.3[0.2 to 145.2], days 8 to 14; 32.6[3.5 to 168.9], days 15 to 21; 36.5[1.8 to 200.9], days 22 to 28; 57.9[5.1 to 209.9], day >28; anti-NC AU/ml: 2.4[0.1 to 138.2], days 1 to 7; 15.2[0.1 to 219.8], days 8 to 14; 61.7[0.1 to 201.7], days 15 to 21; 103.5[0.1 to 236.2], days 22 to 28; 82.4[0.1 to 200.2], day >28; Fig. 2A], whereas virus-specific IgA titers increased during the first 3 weeks post-symptom onset, then dropped, and were undetectable 1 month after recovery (anti-RBD AU/ml: 3.7[0.1 to 69.6], days 1 to 7; 5.2[0.1 to 166.3], days 8 to 14; 9.5[0.1 to 58.5], days 15 to 21; 7.3[0.2 to 149.9], days 22 to 28; 3.1[0.1 to 26.4], day >28; anti-NC AU/ml: 0.1[0.1 to 153.7], days 1 to 7; 3.9[0.1 to 158.2], days 8 to 14; 25.5[0.1 to 128.2], days 15 to 21; 6.9[0.1 to 116.6], days 22 to 28; 0.1[0.1 to 11.9], day >28; Fig. 2A). These results suggest that anti–SARS-CoV-2 IgA testing may improve early COVID-19 diagnosis, but serum testing more than 28 days after the onset of symptoms may only reliably detect IgG antibodies.

### Early SARS-CoV-2–specific IgA response is not associated with COVID-19 severity

We explored the relationship between virus-specific serum Ig titers and the clinical course of patients with COVID-19. We focused the analysis on early serum samples obtained no later than 10 days after the onset of clinical symptoms. A composite end point was used to define severe COVID-19 in patients that were initially admitted to the same intensive care unit (ICU) internal medicine ward and included the following criteria: transfer to the ICU, use of oxygen therapy by nasal cannula above 5 liters/min, high-flow nasal cannula oxygen therapy, invasive or noninvasive mechanical ventilation, severe acute confusional state, acute renal failure, and death. Together, early virus-specific IgG, IgM, and IgA titers were not markedly different in severe patients compared with nonsevere patients (table S4).

### Serum IgA is a potent and early SARS-CoV-2–neutralizing agent

**Fig. 3:**
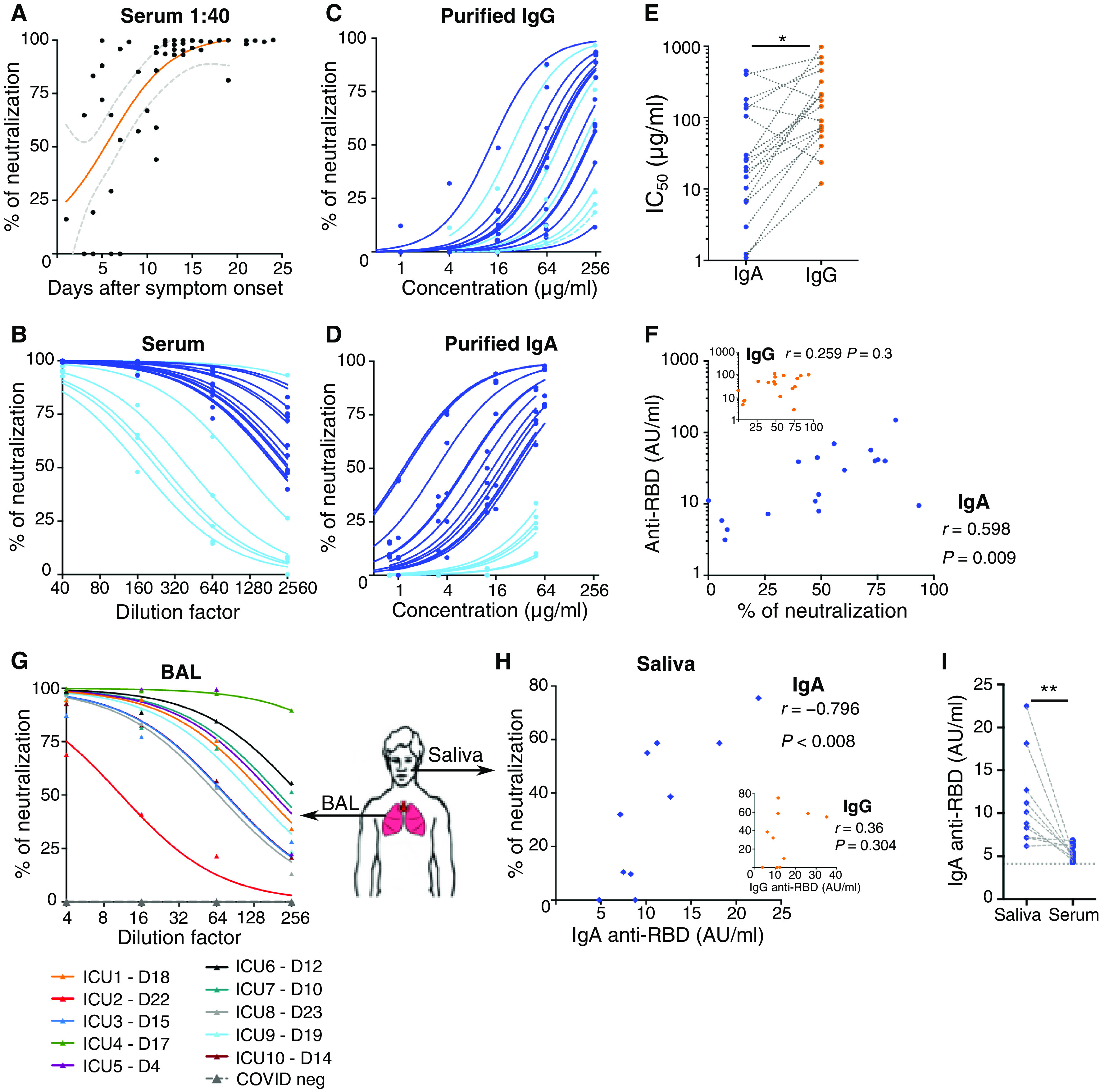
Neutralizing activity of serum, BAL and saliva antibodies to SARS-CoV-2. (**A**) Neutralizing activity of 52 sera (dilution of 1:40) from 38 SARS-CoV-2–infected patients (see clinical characteristics in table S1) was determined using a pseudovirus neutralization assay. Orange curve represents significant sigmoidal interpolation (*P* = 0.0082). Gray dotted curves represent 95% confidence intervals. (**B**) Neutralizing activity of 18 sera measured by pseudovirus neutralization assay at different indicated dilutions. Samples used for this analysis were collected between days 6 and 24 after symptom onset. Light blue color corresponds to samples with low IgA neutralization potential. (**C**) Neutralizing activity of purified IgG was measured at indicated concentrations from 18 sera collected between day 6 and day 24 post-symptom onset. Curves were drawn according to nonlinear regression. Light blue color corresponds to samples with low IgA neutralization potential. (**D**) Neutralizing activity of purified IgA from paired samples in (C). Light blue color corresponds to samples with low IgA neutralization potential. (**E**) Paired purified IgA and IgG IC_50_ values in samples tested in (C) and (D). *P* value was calculated using Wilcoxon test (*P < 0.05). (**F**) Comparison of serum anti-RBD IgA (main panel) or IgG (inset) levels measured by photonic ring immunoassay with neutralizing capacity of corresponding purified isotypes measured by pseudovirus neutralization assay. Spearman coefficient (*r*) and *P* value (*P*) are indicated. (**G)** Neutralizing activity of bronchoalveolar lavages (BALs) collected in 10 SARS-CoV-2 patients between days 4 and 23 after symptom onset (clinical characteristics are detailed in table S5). Indicated BAL dilutions were tested using pseudovirus neutralization assay. BALs obtained from SARS-CoV-2–negative patients (*n* = 3) showed no neutralization activity (dotted gray lines). Each colored line represents one patient. (**H**) Neutralizing activity and anti-RBD IgA levels (both tested at a dilution of 1:4) of saliva collected in 10 SARS-CoV-2 patients between days 49 and 73 after symptom onset. *r* and *P* are indicated. (**I**) Anti-RBD levels in paired saliva and serum from patients tested in (H). *P* value was calculated using Wilcoxon test (**P < 0.01).

We sought to determine the contribution of each of the IgG and IgA isotypes to virus neutralization. We assessed the neutralizing capacity of serum antibodies using a pseudoneutralization assay. We tested the neutralization potential of serum at a dilution of 1:40, which rapidly increased during the course of disease and plateaued by day 10 post-symptom onset (Fig. 3A). Pseudovirus neutralizing activity varied considerably between patients, with half-maximal inhibitory concentration (IC_50_) values ranging from 1:169 to 1:16,189 serum dilution (Fig. 3B). We compared the pseudotyped particle entry inhibition assay with a whole-virus neutralization assay based on the utilization of a live SARS-CoV-2 field isolate (fig. S5A) and found a strong correlation between results obtained using these two assays (*r* = 0.88, *P* < 0.0001), which is consistent with other published findings (*20*, *21*).

We then sought to define the respective contributions of the dominant antibody isotypes to virus neutralization by using purified IgA and IgG fractions from the sera of 18 patients (fig. S5B) that were tested in parallel using the pseudovirus neutralization assay (Fig. 3, C and D). IgA preparations were more potent in their neutralization compared with paired IgG (Fig. 3, C to E). We observed that serum samples with low neutralization potential (light blue curves in Fig. 3B) also had low IgA-based neutralizing activity (corresponding light blue curves in Fig. 3D) and confirmed that serum neutralization potential was only associated with anti-RBD IgA content and not anti-RBD IgG (Fig. 3F). IgA neutralization potential correlated directly with anti-RBD IgA serum titers (*r* = −0.88, *P* < 0.0001; fig. S5C) but not with anti-NC IgA titers (*r* = 0.14, *P* = 0.58; fig. S5D), suggesting that the IgA neutralization potential is closely linked to RBD binding. IgG is about five times more abundant in serum than IgA, but, as shown, purified IgA fractions had about seven times lower IC_50_ values as compared with purified IgG ([minimum to maximum]; IgA IC_50_ [1.1 to 454.9] versus IgG IC_50_ [11.9 to 982.4], *n* = 18; Fig. 3E). In addition, the more efficient neutralization potential of IgA compared with IgG cannot be explained by an avidity effect, because both purified antibody preparations were monomeric (fig. S5B).

These results suggest that IgA contributes more than IgG to serum neutralization potential in the early phase of the infection. There are exceptions to this rule, such as patients #2 and #3, who both presented with very low anti-RBD IgA levels (below 11 AU/ml between days 6 and 18) but instead mounted an early serum IgM response (fig. S5E).

### Mucosal IgA is a SARS-CoV-2–neutralizing agent

These observations detail the respective contributions of IgA and IgG to systemic humoral immunity. However, the main SARS-CoV-2 targets are lung epithelial cells (*22*, *23*), and mucosal immunity differs from systemic immunity. To assess local mucosal immunity, saliva and bronchoalveolar lavage (BAL) samples were also tested. IgG concentrations were consistently higher than IgA in the tested BAL samples, except for one patient (fig. S5F), but IgA concentrations were higher than IgG in saliva (median[minimum to maximum]; IgA 392[92 to 1200] versus IgG [<70 to 130] μg/ml; *n* = 10; fig. S5G). Consistent with these findings, previous reports demonstrated an increased IgG:IgA ratio along the respiratory tract, as measured from the nasal compartment to the lungs (*24*, *25*). In contrast to serum-purified IgA, both monomeric and dimeric IgA were observed in bronchoalveolar fluids (fig. S5H). BAL samples were harvested at various times, including sampling as early as 4 days after symptom onset, and pseudovirus neutralization activity was detected in most samples (Fig. 3G). Anti-RBD IgA were detected in most BAL samples, whereas anti-RBD IgG were above the threshold of positivity in only half of samples (fig. S5I), suggesting that IgA may contribute more than IgG to SARS-CoV-2 neutralization in the lung. Although saliva samples were collected after day 49 post-symptom onset, 8 of the 10 samples neutralized SARS-CoV-2–pseudotyped viral particles, albeit with notable interindividual variability (Fig. 3H). Pseudovirus neutralization activity directly correlated with anti-RBD IgA titers (*r* = −0.796, *P* < 0.008; Fig. 3H) but did not correlate with anti-RBD IgG titers, although anti-RBD IgG were above the threshold of detection (Fig. 3H). Anti-RBD IgA were consistently more abundant in saliva than in serum (9.5[6.2 to 22.5] versus 5.2[4.3 to 6.8], *P* = 0.0039; Fig. 3I), suggesting that SARS-CoV-2–specific IgA may persist longer at mucosal sites compared with peripheral blood in hospitalized COVID-19 patients. Observations at later time points (days 189 to 230 post-symptoms) in a series of ambulatory individuals who had experienced pauci-symptomatic COVID-19 revealed no detectable neutralization activity in saliva (fig. S5J).

## DISCUSSION

Our data highlight the potency of IgA in the early stage of COVID-19 disease at various body sites through the analysis of blood, BAL, and saliva. We show that SARS-CoV-2 neutralization is more closely correlated with IgA than IgM or IgG in the first weeks after symptom onset. Our own results do not directly imply that monomeric IgA would be inherently more neutralizing than monomeric IgG. However, published works already suggest that SARS-CoV-2–specific IgA and IgG responses are qualitatively different. In a recent study based on the same variable SARS-CoV-2–specific antibody domain, but expressed as IgA or IgG, Ejemel *et al*. (*26*) showed that the IgA monomer had significantly enhanced neutralization potency over its IgG equivalent. It is proposed that the increased flexibility and longer hinge of IgA1, relative to IgG (*27*), would be more favorable to interactions between the IgA monomer and the SARS-CoV-2 spike trimer. Previous studies in influenza- and HIV-specific antibodies have reported similar observations (*28*).

Another possibility is that IgA may be more broadly cross-reactive against various human coronaviruses, as suggested from the extensive analysis of memory B cells of a survivor of the 2003 SARS-CoV outbreak (*29*). It is also possible that the maturation of the systemic IgG response may be slightly delayed compared with the mucosal IgA response. Our results show that serum IgA, particularly anti-RBD IgA, is detected earlier compared with IgG, and that the marked plasmablast expansion that following SARS-CoV-2 infection is dominated by IgA-secreting cells (Fig. 1D). The time to positivity against RBD is markedly shorter for IgA than for IgG (fig. S4B), and serum neutralization potential is associated with anti-RBD IgA isotype antibodies (Fig. 3F). We also observed a rapid decline in SARS-CoV-2–specific IgA serum levels, thereby bringing into question the long-term efficacy of this marked first wave response. In convalescent individuals, plasma SARS-CoV-2–specific IgA monomers were found to be twofold less potent in neutralization assays than IgG equivalents (*30*). It is also possible that sustained SARS-CoV-2–specific secretory IgA levels are maintained in mucosal secretions, because we detected higher SARS-CoV-2–specific IgA titers in saliva relative to paired serum samples obtained after day 49 post-symptom onset (Fig. 3I). This observation is consistent with the finding that the dimeric form of IgA, which is found in the mucosa, is more potent against authentic SARS-CoV-2 than both IgA and IgG monomers (*30*). However, neutralizing activity was not detectable at later time points (days 189 to 230) in the saliva of 14 individuals who had exhibited mild, ambulatory, COVID-19 (fig. S5J).

Mucosal SARS-CoV-2–neutralizing antibodies may arise from multiple origins. Monomeric plasma IgA antibodies do not bind to FcRn but can reach the airways through an alternative receptor-independent process called transudation, which is more likely to occur in damaged lung tissue (*31*, *32*). A clonal relationship has been shown between serum and mucosal antigen-specific IgA (*33*). We show that monomeric IgA is present in BAL; thus, it is possible that plasma IgA antibodies could exert functions in the lower intestinal track as well. Whereas IgA and IgG may reach the airways and lungs by transudation from plasma, no significant correlation was observed between BAL and serum-specific antibody titers (fig. S5H), suggesting that a part of the SARS-CoV-2 antibody response is generated locally. Recirculating IgA-secreting plasmablasts with a mucosal homing profile (*16*, *34*–*36*) were detected in high numbers in the patients that we studied and are likely to seed the lung/airway interface. IgA-secreting cells can efficiently home to and reside within the mucosa (*37*), and IgA subclass switch recombination can take place in these tissues (*38*) in a T cell–independent manner (*39*). The lack of correlation between plasmablast and T_FH_ cell expansion observed in this study suggests that germinal center–independent induction of IgA is occurring (*40*). Several recently described SARS-CoV-2–neutralizing IgG (*41*, *42*) did not carry somatic mutations typically associated with affinity maturation and T cell help. A molecular and functional characterization of IgA monoclonal antibodies secreted by plasmablasts found in peripheral blood during the first week of symptom onset may shed light on their mutational status. The observations made in our study could provide insight into the observation that the vast majority of children develop mild symptoms or are asymptomatic upon SARS-CoV-2 infection (*43*, *44*), by suggesting that cross-reactive IgA, recently identified in human gut mucosa against other targets than SARS-CoV-2 (*34*, *45*), may be more prevalent in children and/or could be rapidly mobilized in response to infection with SARS-CoV-2.

We confirmed that serum, BAL, and saliva antibodies have SARS-CoV-2 neutralization potential using a pseudoneutralization assay and validated with a viral neutralization assay. It remains to be confirmed whether this response is long-lasting in patients who have experienced more severe disease compared with the ambulatory patients that were studied here. Saliva analysis, potentially based on newly developed digital enzyme-linked immunosorbent assay (ELISA)–based assays, such as the single molecule array (Simoa) (*46*), may represent a convenient way to address this issue in future studies.

In several early serum samples with efficient virus-neutralizing capacity, only anti-RBD IgM was detected at measurable amounts, because neither IgA nor IgG SARS-CoV-2 spike RBD-specific antibodies were above the threshold of detection [patient 2 (P2) day 14 and P3 day 6 post-symptoms; fig. S5E]. This observation suggests that IgM may provide some level of protection as well, but this sequence of detection of IgM first, followed by IgG and IgA, is unusual and not likely to be prevalent. A more typical profile is exemplified by P9, with anti-RBD IgA levels peaking before the appearance of anti-RBD IgG and barely detectable IgM at any of the measured time-points (fig. S5E). Because only virus-specific IgM is detected at early time points in rare cases, it remains to be determined whether all isotypes should be measured during serological diagnosis.

It was recently proposed that high levels of IgA might play a detrimental role in COVID-19 patients (*47*). We compared early IgA levels in patients with subsequent favorable or severe outcomes. We show that early IgA levels were not significantly higher in patients that later deteriorated (table S4). Our results therefore do not sufficiently support the hypothesis that an early IgA response might have a potential negative influence on disease progression.

Our study has several limitations. Given the time frame covered in this study, further longitudinal studies are needed to assess whether local SARS-CoV-2–specific IgA production persists for a longer time in patients recovered from severe COVID-19 than in the pauci-symptomatic individuals that we have been tested at late time points. In addition, it remains to be determined whether secretory antibodies may contribute to a longer-term barrier effect in the nose and lung compared with saliva.

In conclusion, our findings suggest that IgA-mediated mucosal immunity may be a critical defense mechanism against SARS-CoV-2 at the individual level that may reduce infectivity of human secretions and consequently viral transmission as well. This finding may also inform the development of vaccines that induce specific respiratory IgA responses to SARS-CoV-2.

## MATERIALS AND METHODS

### Study design

We performed a single-center study carried out at Assistance Publique-Hôpitaux de Paris, Sorbonne Université. The target population for the main study was composed of adult, SARS-CoV-2–infected patients. Blood samples were obtained in a longitudinal manner from COVID-19 patients admitted at Hôpital Pitié-Salpêtrière over a period of 1 month. Healthy SARS-CoV-2 negative [polymerase chain reaction (PCR) and serology] individuals, were recruited among laboratory staff members. Samples were processed in the Department of Immunology (G.G.), located on the same hospital site. Flow cytometry and serological studies in the same department, aliquots were also shipped at 4°C to the Pasteur Institute for virological studies (P.C. and O.S.). Serum and saliva samples were also obtained at later time points from hospital-discharged patients consulting in the Department of Internal Medicine (Z.A.) of Institut E3M for planned post-COVID clinical checkups. An ancillary study was performed on the saliva samples of 14 ambulatory health care workers that never required hospitalization but had been exposed to SARS-CoV-2, as confirmed by PCR and/or serology.

### Patient recruitment and sample preparation

Fresh blood sample from 135 consecutive adult patients with COVID-19 referred to Institut E3M, Hôpital Pitié-Salpêtrière, Paris were included in the study between 22 March and 24 April 2020 and compared with 20 age- and sex-matched healthy donors (HDs). The diagnosis of COVID-19 was confirmed for 125 patients by SARS-CoV-2 carriage in the nasopharyngeal swab, as measured by real-time reverse transcription PCR (RT-PCR) analysis. The 10 remaining patients had a negative nasopharyngeal SARS-CoV-2 real-time RT-PCR analysis, but they all had clinical symptoms and a chest computed tomography scan highly evocative of COVID-19, and they all tested positive for the presence of serum anti–SARS-CoV-2 antibodies. Demographic and clinical characteristics are detailed in tables S1 to S3. To define a poor course of the disease after the admission and the first serum sampling, we used a composite end point including transfer to ICU, use of oxygen therapy by nasal cannula above 5 liters/min, high-flow nasal cannula oxygen therapy, invasive or noninvasive mechanical ventilation, severe acute confusional state, acute renal failure, and death. BALs were collected from 10 COVID-19 patients hospitalized in ICUs at Hôpital Pitié-Salpêtrière, Paris and compared with 3 COVID-19–negative samples. Demographic and clinical characteristics are detailed in table S5. This study was approved by the local ethical committee of Sorbonne Université (no. 2020-CER2020-21). For all patients, sera were stored immediately after collection at −80°C. Peripheral blood mononuclear cells (PBMCs) were isolated from the blood samples of 38 patients after Ficoll-Hypaque density gradient centrifugation (Eurobio, Courtaboeuf, France) and analyzed immediately. Clinical characteristics of these patients are presented in table S1.

Saliva samples were self-collected by aspiration using a flexible Pasteur pipette (Thermo Fisher Scientific). Roughly 1 to 3 ml of saliva were collected into a sterile urine cup. All saliva samples were stored at 4°C and transported to the research laboratory of the Department of Immunology within 5 hours of sample collection and stored at −80°C before analysis.

### B cell and T cell phenotyping

Phenotyping was assessed on freshly isolated PBMCs stained with a combination of anti-human antibodies (table S6). Intracellular staining was performed on fixed and permeabilized cells (using the FOXP3 Transcription Factor Staining Buffer Kit; eBioscience). Cells were acquired on a BD FACSCanto II flow cytometer (BD Biosciences) and analyzed with FlowJo v10 software (FlowJo, LLC).

### Serological analysis

SARS-CoV-2–specific IgA, IgM, and IgG antibodies were measured in 214 serum samples from 132 patients with the Maverick SARS-CoV-2 Multi-Antigen Serology Panel (Genalyte Inc., USA) according to the manufacturer’s instructions. The Maverick SARS-CoV-2 Multi-Antigen Serology Panel (Genalyte Inc.) is designed to detect antibodies to five SARS-CoV-2 antigens: nucleocapsid, spike S1 RBD, spike S1S2, spike S2, and spike S1, within a multiplex format based on photonic ring resonance technology (*18*, *19*). This automated system detects and measures with good reproducibility (fig. S3) changes in resonance when antibodies bind to their respective antigens on the chip. Combined IgG and IgM antibodies showed 91% sensitivity and 98% specificity. Briefly, 10 μl of each serum sample was added to a sample well plate array containing required diluents and buffers, and the plate and chip were loaded in the instrument for chip equilibration with the diluent buffer to measure baseline resonance. The serum sample was then charged over the chip to bind specific antibodies to antigens present on the chip. The chip was then washed to remove low-affinity binders, and specific antibodies were detected with anti-IgG, anti-IgA, or anti-IgM secondary antibodies. Forty-three sera collected before December 2019 were analyzed to calculate cutoff values. Positivity was defined as a result above the 99th percentile.

### Purification and quantification of IgA and IgG from serum

IgA and IgG were isolated from 18 serum samples diluted in 1× phosphate-buffered saline (PBS) as previously described (*45*). Sera were selected after SARS-CoV-2–specific antibody evaluation. Briefly, serum samples were loaded onto peptide M/agarose or protein G/agarose column (InvivoGen) after column equilibration. Chromatography steps were performed at a flow rate of 0.5 ml/min. Next, 20 column volumes of 1× PBS were used to wash the column. IgA and IgG were then eluted with 5 ml of 0.1 M glycine (pH 2 to 3; Sigma-Aldrich), and pH was immediately adjusted to 7.5 with 1 M tris. PBS (1×) buffer exchange was achieved using Amicon Ultra centrifugal filters (Merck Millipore) through a 100-kDa membrane according to the manufacturer’s instructions. The quantification of IgA and IgG was determined using NanoVue Plus microvolume spectrophotometers. The purity of the IgG and IgA fractions was assessed by ELISA (IgG, IgM, and IgA ELISA quantitation set; Bethyl Laboratories) according to the manufacturer’s recommendations. Undesirable isotypes (IgM and IgA/IgG counterparts) represented less than 1% of the purified Igs. Electrophoresis was then used to detect Ig monomers and dimers. Purified Ig (1 μg) was separated using 4 to 20% polyacrylamide gel (Mini-PROTEAN TGX Stain-Free Precast Gels; Bio-Rad) in native conditions (Laemmli 4×, Bio-Rad). Gels were incubated with Imperial Protein Stain (Thermo Fisher Scientific) and washed five times with water.

### Pseudovirus production and permissive cell line generation

Pseudotyped vectors were produced by triple transfection of 293T cells as previously described (*48*). Briefly, cells were cotransfected with plasmids encoding lentiviral proteins, a luciferase Firefly reporter, and plasmid expressing a codon-optimized SARS-CoV-2 spike (S) gene. Pseudotyped vectors were harvested at day 2 post-transfection. Functional titer [transducing unit (TU)] was determined by quantitative PCR after transduction of a stable human embryonic kidney (HEK) 293T-hACE2 cell line. To generate this cell line, HEK 293T cells were transduced at a multiplicity of infection (MOI) of 20 with an integrative lentiviral vector expressing human angiotensin-converting enzyme 2 (ACE2) gene under the control of the ubiquitin C promoter. Clones were generated by limiting dilution and selected on their permissivity to SARS-CoV-2 S-pseudotyped lentiviral vector transduction.

### Pseudoneutralization assay

Serum dilutions were mixed and coincubated with 300 TUs of pseudotyped vector at room temperature for 30 min. Serum, BAL, or saliva and vector were then diluted in culture medium [Dulbecco’s modified Eagle’s medium–GlutaMAX (Gibco) + 10% fetal calf serum (Gibco) + 1% penicillin/streptomycin (Gibco)]. This mixture was then plated on tissue culture–treated black 96-well plates (Costar) with 20,000 HEK 293T-hACE2 cells per well in suspension. To prepare the suspension, cell flasks were washed with Dulbecco’s PBS (DPBS) twice (Gibco), and a single-cell suspension was made in DPBS + 0.1% EDTA (Promega) to preserve integrity of hACE2 protein. After 48 hours, the medium was removed from each well and bioluminescence was measured using a luciferase assay system (Promega) on an EnSpire plate reader (PerkinElmer).

### Neutralization assay

U2OS-ACE2 GFP1-10 and GFP11 cells, also referred to as S-Fuse cells (*21*), were mixed (1:1 ratio) and plated at 8 × 10^3^ cells per well in a 96-well flat-bottom plate (μClear, #655090) 24 hours before infection. Cells were then infected with SARS-CoV-2 (strain BetaCoV/France/IDF0372/2020) at an MOI of 0.1. At 18 hours post-infection, cells were fixed in 4% paraformaldehyde for 30 min at room temperature, washed, and resuspended in PBS containing Hoechst 33342 (1:10,000). Images were acquired on the Opera Phenix High Content Screening System (PerkinElmer) and analyzed on Harmony High-Content Imaging and Analysis Software.

### Immunoblotting

BALs were concentrated (5×) using a 100-kDa membrane (Amicon Ultra centrifugal filters; Merck Millipore). Samples were diluted with 4× Laemmli sample buffer (Bio-Rad) and heated at 95°C for 5 min. Proteins were separated using 4 to 20% polyacrylamide gel electrophoresis (Mini-PROTEAN TGX Stain-Free Precast Gels; Bio-Rad) for 30 min at 200 V and then transferred to nitrocellulose for Western blot analysis. Human IgA was detected with horseradish peroxidase–conjugated goat anti-human IgA used at a 1:20,000 dilution for 1 hour (Bethyl Laboratories) followed by the addition of enhanced chemiluminescence (ECL) substrate (Clarity Western ECL, Bio-Rad). Chemiluminescence was visualized with a camera system (ImageQuant LAS4000, GE Healthcare). All incubations were in 1× PBS with 5% nonfat milk, and wash steps used 1× PBS with 0.1% Tween 20.

### Statistical analysis

Variables were expressed as the median. Several nonparametric tests were used including the Mann-Whitney *U* test to compare two independent groups, the Wilcoxon test to compare paired values, and the chi-square test to compare antibody positive rates. The Spearman correlation test was used to measure the correlation between two variables. Statistics were corrected for multiple comparisons with Dunn’s test. Significant *P* values are indicated as described: *P < 0.05, **P < 0.01, ***P < 0.001, and ****P < 0.0001. Statistical analysis was performed using GraphPad Prism software, V6 (GraphPad, San Diego).

## SUPPLEMENTARY MATERIALS

stm.sciencemag.org/cgi/content/full/13/577/eabd2223/DC1 Fig. S1 (related to Fig. 1). Intracellular antibody expression in circulating plasmablasts. Fig. S2 (related to Fig. 1). Circulating T_FH_ cells in blood of SARS-CoV-2-infected patients. Fig. S3 (related to Fig. 2). Reproducibility of photonic ring immunoassay to detect anti-RBD and anti-NC antibodies. Fig. S4 (related to Fig. 2). Early detection of anti-RBD antibodies in serum from SARS-CoV-2-infected patients. Fig. S5 (related to Fig. 3). Neutralizing activity of serum, BAL, and saliva from SARS-CoV-2-infected patients. Table S1. Demographics, baseline characteristics, treatment, and outcome of 38 COVID-19 assessed for blood plasmablasts. Table S2. Demographics and baseline characteristics of COVID-19 patients. Table S3. Clinical characteristics, laboratory results, treatment, and outcome of COVID-19 patients. Table S4. Virus-specific IgG, IgM, and IgA titers according to the clinical course of the disease in COVID-19 patients. Table S5. Demographics, baseline characteristics, treatment, and outcome of patients with acute respiratory distress syndrome during the course of COVID-19. Table S6. Human antibodies used for B and T cell phenotyping. Data file S1. Raw data spreadsheet.

## Appendix

View/request a protocol for this paper from *Bio-protocol*.
